# Asymmetrical Artificial Potential Field as Framework of Nonlinear PID Loop to Control Position Tracking by Nonholonomic UAVs

**DOI:** 10.3390/s22155474

**Published:** 2022-07-22

**Authors:** Cezary Kownacki, Leszek Ambroziak

**Affiliations:** Department of Robotics and Mechatronics, Faculty of Mechanical Engineering, Bialystok University of Technology, Wiejska St. 45C, 15-351 Bialystok, Poland; l.ambroziak@pb.edu.pl

**Keywords:** nonholonomic robots, UAV, position tracking, potential field, PID

## Abstract

Precise position tracking plays a key role in formation flights of UAVs (unmanned aerial vehicles) or other applications based on the idea of the leader–following scheme. It decides on the integrity of a formation or increasing the position error when a UAV follows the desired flight path. This is especially difficult in the case of nonholonomic vehicles having limited possibilities of making turns, causing a lack of stability. An asymmetrical artificial potential field (AAPF) is the way to achieve the stability of position tracking by nonholonomic UAVs, but it is only a nonlinear proportional relation to feedback given by a tracking error. Therefore, there can still be a steady-state error or error overshoots. Combining an AAPF with integral and derivative terms can improve the response of control by damping overshoots and minimizing the steady-state error. Such a combination results in a regulator whose properties allow defining it as nonlinear PID. Numerical simulation confirms that integral and derivative terms together with an AAPF create a control loop that can minimize overshoots of the tracking error and the steady-state error and satisfy conditions of asymptotical stability.

## 1. Introduction

Position tracking is one of the most useful features of present unmanned aerial vehicles. It can be adopted in a variety of applications, starting from formation flights [1,2,3],through operations such as moving platforms [4,5], ending with visual feature tracking [6,7], such person tracking [8], which is common in commercial drone solutions (e.g., DJI). In each of them, the precision of keeping the desired position or distance from a moving reference point plays a crucial role. Therefore, most of them focus on the usage of multirotor construction, whose main advantage is the capability of omnidirectional movement. This class of vehicles belongs to holonomic robots [9,10], in which the controllable degrees of freedom are equal to the total degrees of freedom. Omnidirectional maneuverability makes it easier to achieve a smooth tracking of reference points, maintaining control stability. Whereas, in the case of unmanned fixed-wing aircraft, the problem is more difficult and complex due to nonholonomic constraints. Limited maneuverability resulting in minimum turn radius requires applying different approaches. Therefore, the leader–follower formation control scheme is more popular in the case of fixed-wing UAVs (unmanned aerial vehicles) than other methods [3,11,12,13,14]. The leader–follower control scheme assumes that the follower UAV tracks the leader UAV’s position at a predefined safe distance, which is, in fact, the predefined set-point value of the tracking error. This eliminates situations when it changes its sign unintentionally, limiting the range of allowable heading angle change. Other methods used in fixed-wing formation flights are based on structure and motion synchronization [15], model predictive control algorithms applied to track the reference trajectory [16], and artificial potential fields [14]. The artificial potential field (APF) is one of the methods that allow achieving optimal flight path, guiding a UAV among obstacles towards a mission target [17]. Mathematical nonlinear functions used to create the APF and related velocity vector fields possess satisfactory requirements to be candidates for Lyapunov functions [18]. Lyapunov functions are scalar functions that guarantee the asymptotical stability of equilibrium of an ordinary differential equation. Therefore, in most cases of APF methods, UAV guidance applies a velocity vector field that is symmetrical around the point where the potential function reaches its minimum. Such a kind of potential field fails in the case of a nonholonomic vehicle [19]. The artificial potential field (APF) combined with nonholonomic constraints of a UAV causes instability as the result of violent heading and airspeed changes due to the inertia in a UAV’s response to changing relative position. Therefore, novel formulas of a potential field solve this stability issue [19,20]. The idea is to bound the allowable range of heading angles and airspeed which corresponds to the nonholonomic constraints of fixed-wing UAV. This requires an adequate potential field in which gradients smoothly target in one specified direction. One of the solutions in the field of artificial potential fields satisfying that requirement is described in [20] and its further evolution in [21]. The solution is called a method of an asymmetrical artificial potential field (AAPF). The main feature of the proposed method of an AAPF is an asymmetrical distribution of velocity vector field in relation to a line of instantaneous direction of the tracked point movement. Although there is proof that such an AAPF provides asymptotically stability of position tracking, it does not include resistance to external factors, such as wind gusts which disturb the dynamics and precision of position tracking [20]. This is because potential artificial fields define only a nonlinear geometrical relationship between velocity vector and the position of a UAV relative to the tracked point (i.e., tracking error) without including data about how it changes in time. Hence, AAPF can be treated only as a nonlinear function of tracking error, which acts as a nonlinear proportional term of a PID regulator. This article shows that it is possible to extend the definitions of AAPF and related velocity vector field with derivative and integral terms of PID controller, which will be defined also as nonlinear functions of position tracking error. This approach results in the novel form of nonlinear PID controller which is perfectly suited to control dynamics of position tracking by nonholonomic UAVs. We can consider it as geometrical nonlinear PID control like in [22], but in contrast, it inherits properties of the AAPF, which guarantees asymptotic stability for nonholonomic vehicles. There are definitions of novel nonlinear integral (I) and derivative (D) elements of PID controllers with position tracking error as input and the AAPF as the nonlinear proportional element in this article. The usage of D and I terms with the AAPF makes the guidance of position tracking able to respond to both dynamics and steady-state tracking error, still providing the asymptotical stability in the case of nonholonomic UAVs [20]. We carried out numerical simulations and it was proved that the effectiveness of the proposed position tracking approach is better than in the case of the approach based only on an AAPF, especially if a UAV flight is during windy conditions. Therefore, highlighting the advantages of the proposed fusion of AAPF properties with the benefits of a PID controller in reference to the standalone approach AAPF applied to the problem of position tracking by nonholonomic UAVs is the main contribution and the novelty of the article. Moreover, the AAPF approach is an advantage itself in reference to other known methods of position tracking. The following part of the article has five sections. In Section 2, there is a description of the asymmetrical potential field presented as nonlinear proportional terms independently for each axis of the reference frame. Section 3 and Section 4 present respectively definitions of I and D terms, which together with an asymmetrical potential field create a nonlinear PID loop, which determines the novelty of the proposed approach. Section 5 presents numerical simulations and their results. The article ends with conclusions (Section 7) based on simulations results presented in Section 6. In Table 1 there is a list of symbols and acronyms used throughout the article.

## 2. Artificial Asymmetrical Potential Field as Nonlinear Proportional Term

**Figure 1:**
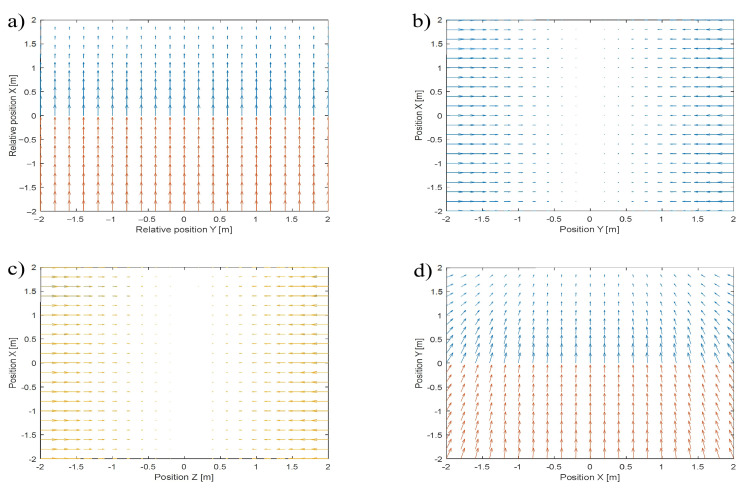
Velocity vector subfields, components of the velocity vector field defined around: (**a**) axis *x*, a cross-section in the plane *x*-*y*, (**b**) axis *y*, a cross-section in the plane *x*-*y*, (**c**) axis *z*, a cross-section in the plane *x*-*z*, (**d**) the velocity vector field based on APF as a superposition of subfields (**a**–**c**) (a cross-section in plane *x*-*y*).

To achieve global asymptotical stability, the artificial asymmetrical potential field approach decouples a position control in longitudinal and lateral directions. In the longitudinal direction, the control is based on nonlinear asymmetrical control of velocity which increases or decreases according to the sign of position tracking error in axis *x*, i.e., along the direction of a reference point movement. Therefore, on both sides of axis *y*, there are areas named an acceleration zone, when the position tracking error in axis *x* is positive, and a deceleration zone, when the error is negative. The *x*-axis component of a velocity vector is an output of the velocity control. In turn, control in lateral directions must align the UAV’s heading and pitch angles to the heading and pitch angles of the reference point. A control of heading and pitch angles assumes a symmetrical nonlinear relation between tracking errors in axes y and z, and velocity in these axes. The control of heading and pitch angles defines components of the velocity vector respectively in axis *y* and axis *z*. The decoupled position control for each axis based on the AAPF is shown in Figure 1a–c, where the components of the velocity vector field around and respected to each axis are presented as cross-sections of velocity vector subfields in planes *x*-*y* or *x*-*z*. Figure 1d presents the superposition of all subfields composing the resultant velocity vector field based on the AAPF, shown as cross-section in the plane *x*-*y*.

In Figure 1a,d, velocity vector fields are in different colors to indicate areas of the acceleration and deceleration zones. Figure 1 explains the global asymptotical stability of position tracking based on the AAPF approach. A potential function for the velocity vector field in Figure 1a reaches its minimum equal to zero along the line of axis *y* (1). Similarly, potential functions for the velocity vector fields in Figure 1b,c have minimums equal to zero along axis *x*. Therefore, the sum of all potential functions giving the AAPF from Equation (1) has a minimum located exactly at the point of intersection of two lines of axes *x* and *y*. If the minimum equals zero and the value of the function for each value of *x*, *y*, and *z*, is greater than zero, the function will satisfy conditions for Lyapunov function and global asymptotical stability [20].

The definition of asymmetrical potential function $U_{i}^{S}\left(x,y,z\right)$ formulated (Figure 2b) in [20] is as follows:

(1) $$U_{i}^{S}\;\left(x,y,z\right)=\left\{\begin{array}{cc} V_{L}\cdot \arctan\left(\alpha \cdot x\right)+\frac{1}{3}\cdot \gamma \cdot \left|y^{3}\right|+\frac{1}{3}\cdot \gamma \cdot \left|z^{3}\right| & x\geq 0\\ V_{L}\cdot \left|\alpha \cdot x\right|+\frac{1}{3}\cdot \beta \cdot \left|x^{3}\right|+\frac{1}{3}\cdot \gamma \cdot \left|y^{3}\right|+\frac{1}{3}\cdot \gamma \cdot \left|z^{3}\right| & x<0 \end{array},\right.$$

where $V_{L}$—airspeed of the tracked virtual point, α,β,γ—coefficients regulating the slope of the potential function, i.e., in the forward longitudinal direction, (x⩾0)—the rate of deceleration, in the backward direction, (x<0)—the rate of acceleration, and in both perpendicular directions, lateral and vertical, (*y* and *z*)—respectively, rate of heading and pitch.

**Figure 2:**
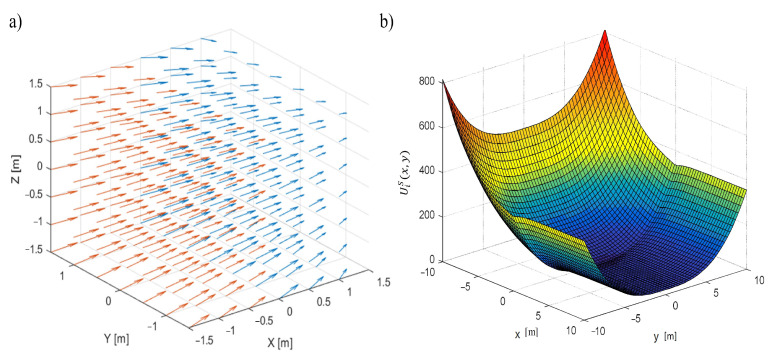
(**a**) The velocity field $V_{i}^{S}\left(x,y,z\right)$, the zone indicated with a red vector is the zone of acceleration, the zone with a blue vector is the zone of deceleration, (**b**) potential field $U_{i}^{S}$ as function of *x* and *y*, *z* is constant [20].

The gradient of the potential function $U_{i}^{S}$ used to create control velocity vector field $V_{i}^{S}$ (Figure 2) is as follows [20]:

(2) $$\nabla U_{i}^{S}\left(x,y,z\right)=\left\{\begin{array}{cc} \left[\frac{\alpha \cdot V_{L}}{1+{\left(\alpha \cdot x\right)}^{2}},\gamma \cdot sgn\left(y\right)\cdot y^{2},\gamma \cdot sgn\left(z\right)\cdot z^{2}\right] & x\geq 0\\ \left[sgn\left(x\right)\cdot \left(\alpha \cdot V_{L}+\beta \cdot x^{2}\right),\gamma \cdot sgn\left(y\right)\cdot y^{2},\gamma \cdot sgn\left(z\right)\cdot z^{2}\right] & x<0 \end{array},\right.$$

Equation (2) defines velocity vector field $V_{i}^{S}$ (Figure 2a) based on gradient $\nabla U_{i}^{S}$ [21].

(3) $$V_{i}^{S}\left(x,y,z\right)=\left[\begin{array}{c} \nabla U_{i}^{S}\left(x\right)\\ -\nabla U_{i}^{S}\left(y\right)\\ -\nabla U_{i}^{S}\left(z\right) \end{array}\right]\cdot D\left(\dot{\Psi_{L}}\right)=\left[\begin{array}{c} \nabla U_{i}^{S}\left(x\right)\\ -\nabla U_{i}^{S}\left(y\right)\\ -\nabla U_{i}^{S}\left(z\right) \end{array}\right]\cdot \left[\begin{array}{ccc} cos\left(\varepsilon \cdot \dot{\Psi_{L}}\right) & sin\left(\varepsilon \cdot \dot{\Psi_{L}}\right) & 0\\ -sin\left(\varepsilon \cdot \dot{\Psi_{L}}\right) & cos\left(\varepsilon \cdot \dot{\Psi_{L}}\right) & 0\\ 0 & 0 & 1 \end{array}\right],$$

where $\dot{\Psi_{L}}$—rate of heading angle of the tracked virtual point, $\nabla U_{i}^{S}\left(x\right)$—component of gradient $\nabla U_{i}^{S}$ in axis *x*,$\nabla U_{i}^{S}\left(y\right)$—component of gradient $\nabla U_{i}^{S}$ in axis *y*, $\nabla U_{i}^{S}\left(z\right)$—component of gradient $\nabla U_{i}^{S}$ in axis *z*, and ϵ—a gain coefficient having the meaning of time constant related to the inertia of response to tracked point turn.

Rewriting Equation (3) into Equation (4) allows splitting the velocity vector field into subfields from Figure 1a (axis *x*), Figure 1b (axis *y*), and Figure 1c (axis *z*).

(4) $$V_{i}^{S}\left(x,y,z\right)=\left(\begin{array}{c} \underset{︸}{\left[\begin{array}{c} \nabla U_{i}^{S}\left(x\right)\\ 0\\ 0 \end{array}\right]}\\ P-axis\;x \end{array}+\begin{array}{c} \underset{︸}{\left[\begin{array}{c} 0\\ -\nabla U_{i}^{S}\left(y\right)\\ 0 \end{array}\right]}\\ P-axis\;y \end{array}+\begin{array}{c} \underset{︸}{\left[\begin{array}{c} 0\\ 0\\ -\nabla U_{i}^{S}\left(z\right) \end{array}\right]}\\ P-axis\;z \end{array}\right)\cdot D\left(\dot{\Psi_{L}}\right),$$

Each vector specified for the associated axis in Equation (4) is a nonlinear function of tracking error in a given axis such as gradient $\nabla U_{i}^{S}\left(x,y,z\right)$ itself. Their lengths are strictly dependent on magnitudes of tracking errors in the related axis and therefore they can be considered as nonlinear proportional terms of PID loops. Let us formulate those PID terms for each axis based on Equation (4).

(5) $$P\left(x\right)=\left[\begin{array}{c} \nabla U_{i}^{S}\left(x\right)\\ 0\\ 0 \end{array}\right],\;P\left(y\right)=\left[\begin{array}{c} 0\\ -\nabla U_{i}^{S}\left(y\right)\\ 0 \end{array}\right],\;P\left(z\right)=\left[\begin{array}{c} 0\\ 0\\ -\nabla U_{i}^{S}\left(z\right) \end{array}\right],$$

Proportional control based only on relative position is insufficient for a precise and stable position tracking control under the influence of disturbances like wind gusts. Therefore, an integral term should extend the definition from (4). This allows minimizing the steady-state error. Nonlinear integral terms for each axis are in the next section.

## 3. Nonlinear Integral Term of PID Control Loop

The general purpose of the integral term of the PID control loop is to minimize the steady-state error. It amplifies the response of the control system as a function of the integral of error which grows with time in the case of the presence of the steady-state error. The side effect is the deterioration of stability. In the case of position tracking, it compensates for the permanent influence of wind and improves the precision of the control. The AAPF is a nonlinear function of tracking error and therefore there are two ways to implement integral terms. At first, they are simple additions to the proportional term as it is in definitions of traditional PIDs and PIs. In the second, they create a uniform nonlinear function of PI controller depending on constraints of the output and specific properties of the control that excludes a simple sum of P and I terms [23,24,25]. In the case of position tracking based on the AAPF, the implementation of integral terms should be independent of the longitudinal and traversal directions, and the zones of the acceleration and deceleration. This is because of the deceleration zone, where the steady-state error in the axis *x* should decrease the UAV’s velocity in this axis smoothly starting from a maximum value, i.e., reference position velocity. Reversely, in the acceleration zone or along axes y and z it should increase the velocity respectively in each axis up to the speed limit of the UAV. Therefore, definitions of integral terms for each axis are different. For axis *x* a nonlinear PI controller is as follows:

(6) $$PI\left(x\right)=\left[\begin{array}{c} f_{PI}\left(x,I\left(x\right)\right)\\ 0\\ 0 \end{array}\right],$$

where $f_{PI}$ is nonlinear function based on $\nabla U_{i}^{S}\left(x\right)$ replacing coefficients and by sums of them with the integral I(x) amplified by $\delta_{Ix1}$ and $\delta_{Ix2}$.

(7) $$f_{PI}\left(x,I\left(x\right)\right)=\left\{\begin{array}{cc} \frac{\alpha \cdot V_{L}}{1+\left(\alpha +\delta_{Ix1}\cdot \left|I\left(x\right)\right|\right)\cdot x^{2}} & x\geq 0\\ sgn\left(x\right)\cdot \left(\alpha \cdot V_{L}+\left(\beta +\delta_{Ix2}\cdot \left|I\left(x\right)\right|\right)\cdot x^{2}\right) & x<0 \end{array},\right.$$

Comparing Equations (6) and (7) with (5), it can be noticed that if the integral I(x) equals zero, the following identity is true $f_{PI}\left(x,I\left(x\right)\right)=\nabla U_{i}^{S}\left(x\right)$. While for nonzero values of the integral I(x), the strength of the acceleration or deceleration effect is proportional to I(x). The purpose of integral terms is to overcome the disturbance impacts on position tracking. Therefore, the integral I(x) should compensate for the steady-state error in axis *x*, only when the source of disturbance and a UAV are on the opposite sides of the tracked point. If a UAV is on the same side of the tracking point where the wind gust comes from, the integral will only amplify the wind effect, causing instability. Hence, the integral I(x) has the meaning for position tracking only when its sign is the same as the sign of the tracking error. Hence, a definition of the integral I(x) is as follows:

(8) $$I\left(x\right)=\left\{\begin{array}{cc} \int_{0}^{\tau }xdt & \left(x\geq 0\cap \int_{0}^{\tau }xdt\geq 0\right)\cup \left(x<0\cap \int_{0}^{\tau }xdt<0\right)\\ 0 \end{array},\right.$$

**Figure 3:**
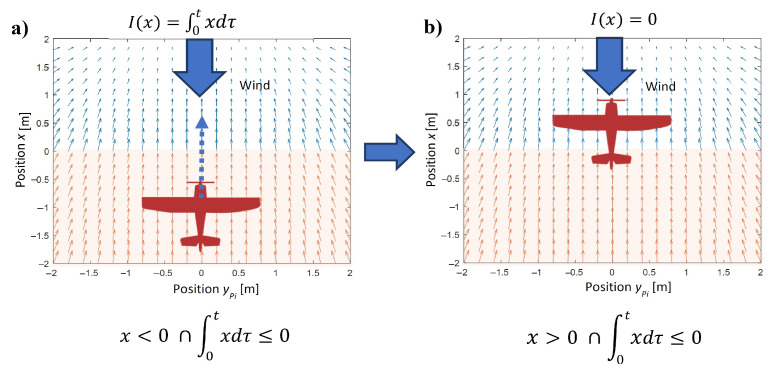
Switching a value of I(x) according to the relative position of a UAV in respect to the tracked point and direction of wind gust. (**a**) the value of I(x) is different than zero, (**b**) the value of I(x) equals zero.

Figure 3 explains the definition of I(x) and its usage in the control.

As mentioned above in lateral directions, integral terms should increase components of velocity vector respectively in axes *y* and *z* according to the tracking errors in each axis. There are no constraints on velocity in these axes related to the velocity of the tracked point as it is in the case of the acceleration zone. Therefore, integral terms simply sum to the proportional terms. Definitions of nonlinear PI controllers for *y* and *z* axes are as follows:

(9) $$PI\left(y\right)=\begin{array}{c} \underset{︸}{\left[\begin{array}{c} 0\\ -\nabla U_{i}^{S}\left(y\right)\\ 0 \end{array}\right]}\\ P \end{array}+\begin{array}{c} \underset{︸}{\left[\begin{array}{c} 0\\ \delta_{Iy}\cdot I\left(y\right)\\ 0 \end{array}\right]}\\ I \end{array},PI\left(z\right)=\begin{array}{c} \underset{︸}{\left[\begin{array}{c} 0\\ 0\\ -\nabla U_{i}^{S}\left(z\right) \end{array}\right]}\\ P \end{array}+\begin{array}{c} \underset{︸}{\left[\begin{array}{c} 0\\ 0\\ \delta_{Iz}\cdot I\left(z\right) \end{array}\right]}\\ I \end{array},$$

where I(y)—the integral of tracking error in axis *y*, I(z)—the integral of tracking error in axis *z*, $\delta_{Iy}$, and $\delta_{Iz}$—integrals’ coefficients.

**Figure 4:**
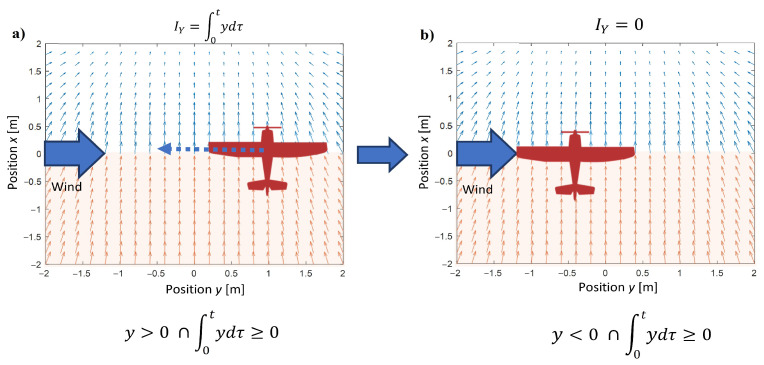
Switching a value of I(y) according to the relative position of a UAV in respect to the tracked point and the direction of wind gust. (**a**) the value of I(y) is different than zero, (**b**) the value of I(y) equals zero.

Similar to Equation (8), the action of integrals terms in the control for axes *y* and *z* should be correlated to the direction of wind and UAV’s relative position (Figure 4). Hence, their definitions are identical:

(10) $$I\left(y\right)=\left\{\begin{array}{cc} \int_{0}^{\tau }ydt & \left(y\geq 0\cap \int_{0}^{\tau }ydt\geq 0\right)\cup \left(y<0\cap \int_{0}^{\tau }ydt<0\right)\\ 0 \end{array},\right.$$

(11) $$I\left(z\right)=\left\{\begin{array}{cc} \int_{0}^{\tau }zdt & \left(z\geq 0\cap \int_{0}^{\tau }zdt\geq 0\right)\cup \left(z<0\cap \int_{0}^{\tau }zdt<0\right)\\ 0 \end{array},\right.$$

The next step is to prepare and define derivative terms, which are important elements in the position tracking control. They improve the dynamics of the control and a response to a rapid change of the tracking error, to reduce relative position overshoots.

## 4. Nonlinear Derivative Term of PID Control Loop

The derivative term of the PID controller is useful in minimizing overshoots of a system’s error and regulation time, which improves response and stability in transient state. Therefore, extending position tracking control with derivative terms should result in the improvement of response to sudden position change due to disturbances such as wind gusts. The position tracking control based on the AAPF defines derivative terms in the same manner as integral terms. In axis *x* for x>0, i.e., in the acceleration zone, a derivative term must be inside the nonlinear function of gradient $\nabla U_{i}^{S}\left(x\right)$ to keep the limit of UAV’s maximum velocity equals to the velocity of the tracked point in the point of equilibrium. To achieve this, identity $f_{PD}\left(x,D\left(x\right)\right)=f_{PID}\left(x,I\left(x\right),D\left(x\right)\right)=\nabla U_{i}^{S}\left(x\right)$ for x=0 must also be true. Hence, definitions of a nonlinear PID controller for axis *x* and the function $f_{PID}\left(x,I\left(x\right),D\left(x\right)\right)$ is as follows:

(12) $$PID\left(x\right)=\left[\begin{array}{c} f_{PID}\left(x,I\left(x\right),D\left(x\right)\right)\\ 0\\ 0 \end{array}\right],$$

(13) $$f_{PID}\left(x,I\left(x\right),D\left(x\right)\right)=\left\{\begin{array}{cc} \frac{\alpha \cdot V_{L}}{1+\left(\alpha +\delta_{Ix2}\cdot \left|I\left(x\right)\right|+\delta_{Dx1}\cdot D\left(x\right)\right)\cdot x^{2}} & x\geq 0\\ sgn\left(x\right)\cdot \left(\alpha \cdot V_{L}+\left(\beta +\delta_{Ix2}\cdot \left|I\left(x\right)\right|+\delta_{Dx2}\cdot D\left(x\right)\right)\cdot x^{2}\right) & x<0 \end{array},\right.$$

where $\delta_{Dx1}$, $\delta_{Dx2}$—coefficients of derivative term $D\left(x\right)=\frac{dx}{dt}$, $\delta_{Ix1}$, $\delta_{Ix2}$ —coefficients of integral term I(x).

In the case of axes *y* and *z*, derivative terms D added to PI controllers from Equation (9) create definitions of nonlinear PID controllers for these axes:

(14) $$PID\left(y\right)=\begin{array}{c} \underset{︸}{\left[\begin{array}{c} 0\\ -\nabla U_{i}^{S}\left(y\right)\\ 0 \end{array}\right]}\\ P \end{array}+\begin{array}{c} \underset{︸}{\left[\begin{array}{c} 0\\ \delta_{Iy}\cdot I\left(y\right)\\ 0 \end{array}\right]}\\ I \end{array}+\begin{array}{c} \underset{︸}{\left[\begin{array}{c} 0\\ \delta_{Dy}\cdot D\left(y\right)\\ 0 \end{array}\right]}\\ D \end{array},$$

(15) $$PID\left(z\right)=\begin{array}{c} \underset{︸}{\left[\begin{array}{c} 0\\ 0\\ -\nabla U_{i}^{S}\left(z\right) \end{array}\right]}\\ P \end{array}+\begin{array}{c} \underset{︸}{\left[\begin{array}{c} 0\\ 0\\ \delta_{Iz}\cdot I\left(z\right) \end{array}\right]}\\ I \end{array}+\begin{array}{c} \underset{︸}{\left[\begin{array}{c} 0\\ 0\\ \delta_{Dz}\cdot D\left(z\right) \end{array}\right]}\\ D \end{array},$$

where $\delta_{Dy1}$, $\delta_{Dy2}$—coefficients of derivative term $D\left(y\right)=\frac{dy}{dt}$, $\delta_{Dz1}$, $\delta_{Dz2}$—coefficients of derivative term $D\left(z\right)=\frac{dz}{dt}$, $\delta_{Iy}$—a coefficient of integral term I(y), $\delta_{Iz}$—a coefficient of integral term I(z), $\nabla U_{i}^{S}\left(y\right)$, and $\nabla U_{i}^{S}\left(z\right)$—elements of the gradient from Equation (2).

Equations (12)–(15) together formulate a novel nonlinear PID controller dedicated for position tracking in-flight by nonholonomic UAVs such as fixed-wings. Because it applies the AAPF as proportional terms of feedback of position error, it keeps Lyapunov function properties to provide the global asymptotical stability. Extending the AAPF with integral and derivative terms it is possible to reduce control overshoots and steady-state errors. To verify and adjust the coefficients of the nonlinear PID controller, a series of numerical simulations were carried out. The simulation results are in the next section.

## 5. Numerical Simulations

**Figure 5:**
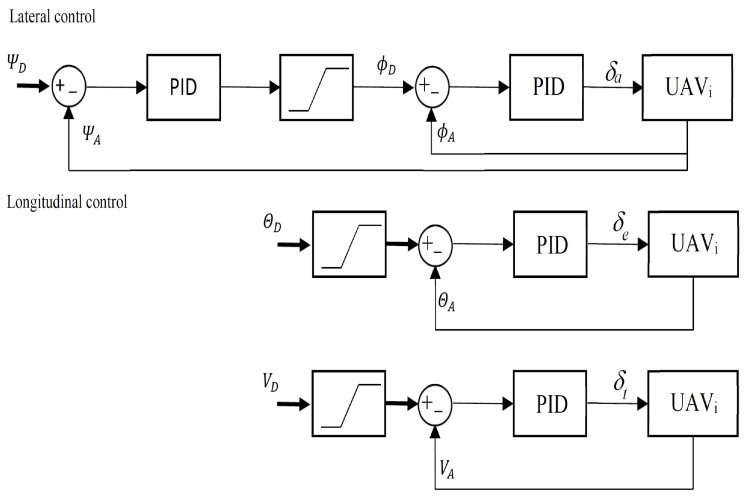
The structure of lateral and longitudinal control of a UAV. $\Psi_{D}$—the desired heading from Equation (16), $\Psi_{A}$—the actual heading, $\phi_{D}$—the desired roll angle, $\phi_{A}$—the actual roll angle, $\delta_{a}$—aileron’s deflection, $\theta_{D}$—the desired pitch angle from Equation (17), $\theta_{A}$ —the actual pitch angle, $\delta_{e}$—elevator deflection, $V_{D}$—the desired airspeed from (18), $V_{A}$—the actual airspeed, and $\delta_{t}$—throttle.

The output of the nonlinear PID controller applied to the control of position tracking is a velocity vector whose length and spatial attitude guide the UAV toward the tracking point. Usually, UAV’s guidance applies loops of lateral and longitudinal control based on three following state variables: heading angle, pitch angle, and airspeed. Outputs of these loops are set-point inputs for inner control loops of deflections of steering elements, i.e., ailerons, elevators, and the throttle of a propeller engine. Therefore, the UAV’s control system has a hierarchical structure, typically based on traditional PID controllers with feedforward terms and gains scheduling. Figure 5 presents the structure of lateral and longitudinal control of a fixed-wing UAV. The nonlinear PID controller of the loop of position tracking is the most outer loop in the structure, hence the velocity vector is the input for the guidance layer. To calculate set-points for heading, pitch, and airspeed loops, it is necessary to apply the following equations [20]:

(16) $$\psi_{D}=atan2\left(PID(y),PID(x)\right),$$

(17) $$\theta_{D}=atan2\left(PID\left(z\right),\sqrt{{PID(x)}^{2}+{PID(y)}^{2}}\right),$$

(18) $$V_{D}=\sqrt{{PID(x)}^{2}+{PID(y)}^{2}+{PID(z)}^{2}},$$

where PID(x) is the nonlinear PID’s output for axis *x* (Equation (12)). PID(y) and PID(z) are outputs of the nonlinear PID respectively for axes *y* and *z* given by Equations (14) and (15).

The UAV’s control system uses saturations on inputs of PID loops of angles of roll, pitch, and airspeed which confine the state-space of UAV’s dynamics to a zone in which the UAV is controllable by onboard electronics. Numerical simulations apply saturation thresholds of these loops’ inputs, typically for small fixed-wing UAVs (wingspan less than 1.5 m and total payload mass than 2 kg), i.e., the limit of roll angle is a range of $\pm 30^{{}^{\circ}}$, the limit of pitch angle is a range of $\pm 15^{{}^{\circ}}$ and for airspeed is a range of 10–20 m/s. To simulate the UAV’s dynamics, numerical simulations use the 6DoF mathematical model of a fixed-wing UAV [26] and discrete wind gust model implementing a wind gust of the standard “1-cosine” shape as it is in the Military Specification MIL-F-8785C. In simulations, the maximum gust magnitudes are 3 m/s and 5 m/s, respectively for the longitudinal and lateral directions. To verify the response of the control to varying wind direction, simulations assume two scenarios, where it changes about $180^{{}^{\circ}}$ in the lateral and longitudinal direction of a flight path. Each scenario verifies the tracking control in each of the directions for different values of coefficients γ, $\delta_{Iy}$, $\delta_{Dy}$ in the first scenario and α, β, $\delta_{Ix1}$, $\delta_{Ix2}$, $\delta_{Dx1}$, $\delta_{Dx2}$ in the second one. Values of $\delta_{Iz}$ and $\delta_{Dz}$ are the same as in sequence $\delta_{Iy}$ and $\delta_{Dy}$.

### 5.1. Lateral Wind Gust Case

**Figure 6:**
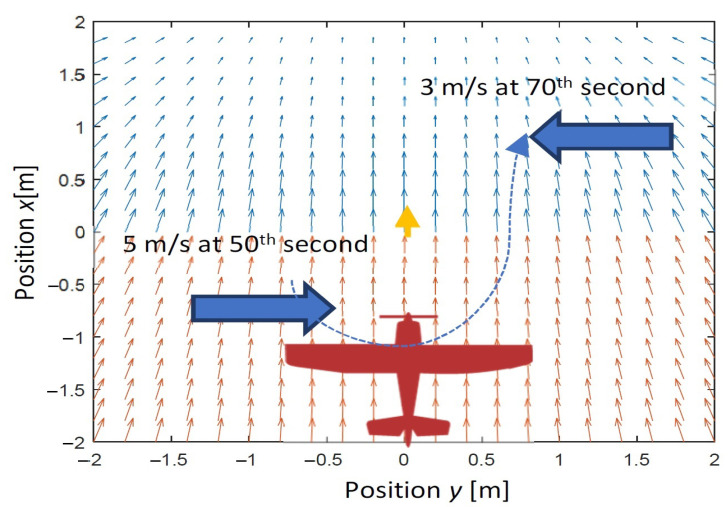
The first scenario of simulations for lateral wind gust case.

The first scenario of simulations assumes that there is a wind gust on the left side of the flight path. It starts at the 50th second of simulations and changes its direction to about $180^{{}^{\circ}}$ at the 70th second. The tracked reference point is moving with a constant speed equal to 15 m/s along a straight line. The maximum speed of wind gust is 5 m/s. The scenario is in Figure 6.

### 5.2. Longitudinal wind Gust Case

**Figure 7:**
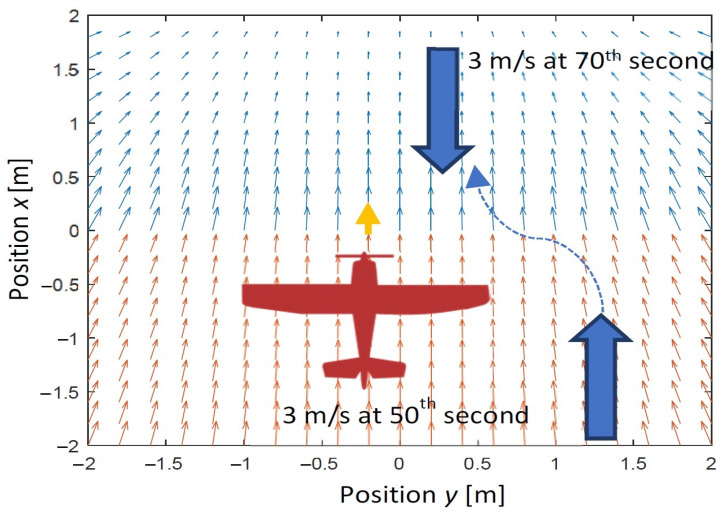
The second scenario of simulations for longitudinal wind gust case.

In the second scenario, there is a backwind gust which also starts at the 50th second of simulations and it changes to a headwind at the 70th second. Similarly, as it is in the first scenario, the tracked reference point is moving straightly along axis *x* and its speed steeply increases to 15 m/s at the beginning of the simulation. The maximum airspeed of the UAV (20 m/s) is greater than the sum of the wind speed (3 m/s) and the speed of the tracked point (15 m/s). In turn, the minimum airspeed of the UAV (10 m/s) is lower than the difference between the wind speed and the speed of the tracked point. These assumptions are obligatory to make the UAV able to achieve relative velocity with respect to the tracked point which is necessary to align position and velocity. The second scenario is in Figure 7.

**Figure 8:**
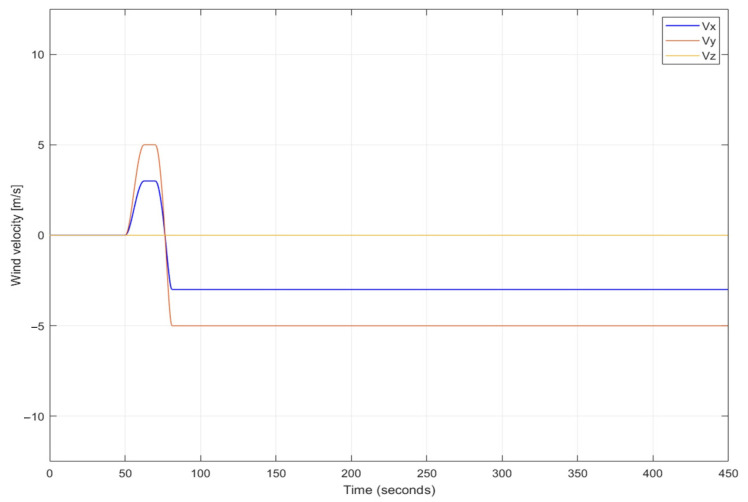
Simultaneous discrete wind gusts along *x* and *y* axes at 50th second and changing its directions to opposite at 75th second.

Figure 8 presents time plots of velocityof simulated discrete wind gusts in the same coordinates frame, in which the UAV tracks a target position. Simulations used the model of a wind gust of the standard “1-cosine” shape as it is in the Military Specification MIL-F-8785C [27,28].

## 6. Results

**Figure 9:**
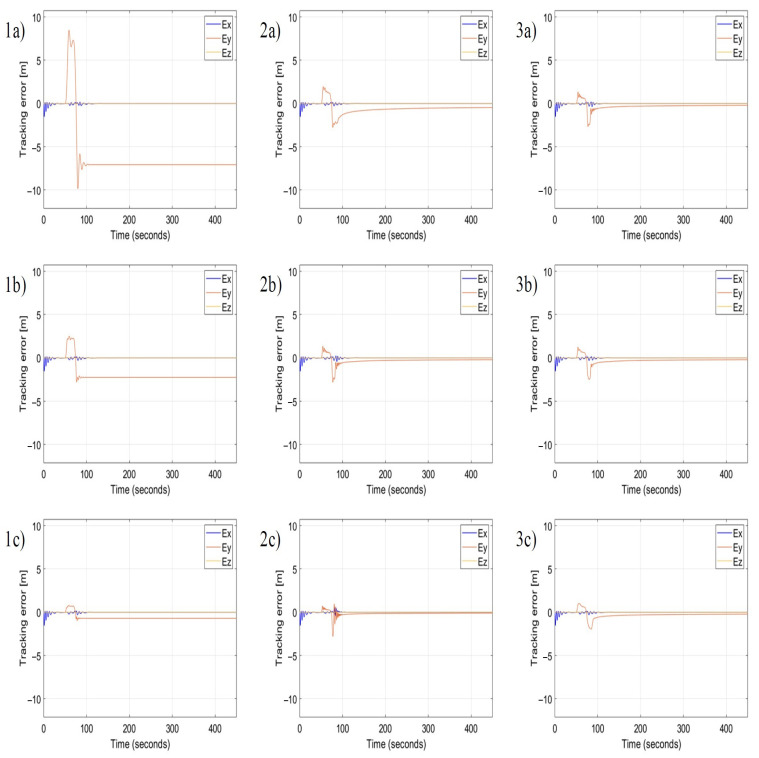
Time plots of position tracking errors for different coefficients γ,$\delta_{Iy}$, $\delta_{Iz}$, $\delta_{Dy}$, and $\delta_{Dz}$ for the nonlinear P, PI, and PID controllers in the case of lateral wind gusts. The applied values of the coefficients are given in Table 2.

Figure 9 presents a comparison of time plots of position tracking errors for the nonlinear P, PI, and PID controllers based on the AAPF in the case of a lateral wind gust. To observe differences in the response of each type of nonlinear controller, the used values for each coefficient of γ, $\delta_{Iy}$, $\delta_{Iz}$, $\delta_{Dy}$, $\delta_{Dzy}$, are as follows: 0.1, 1, and 10.

**Figure 10:**
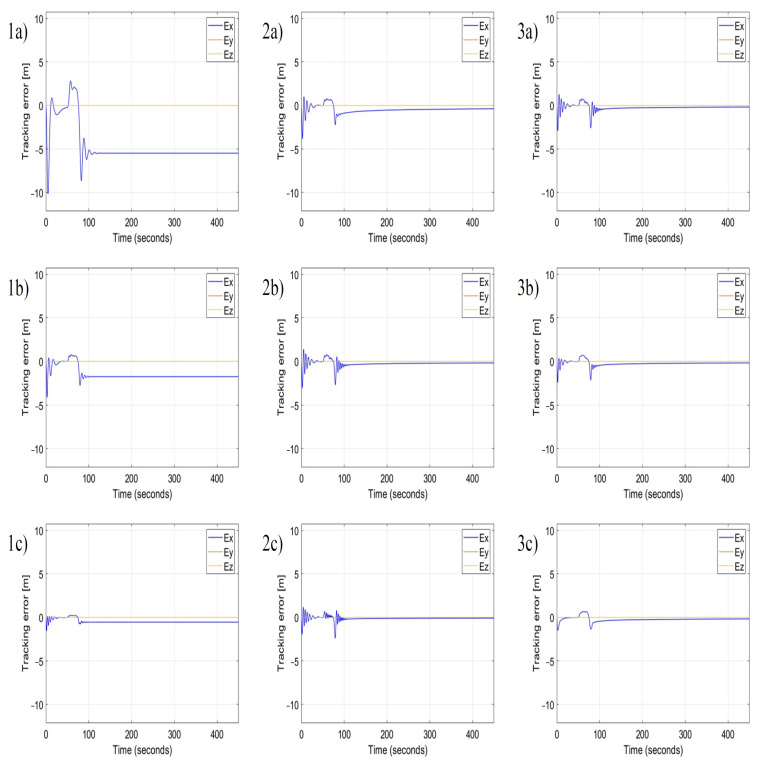
Time plots of position tracking errors for different coefficients α, β, $\delta_{Ix1}$, $\delta_{Ix2}$, $\delta_{Dx1}$ and $\delta_{Dx2}$ for the nonlinear P, PI, and PID controllers in the case of longitudinal wind gusts. Applied values of coefficients are given in Table 3.

In each simulation, the other parameters are constant, i.e., α=β=10, ε=0.3. The longitudinal control of position tracking uses only the nonlinear proportional term P, therefore, the following parameters $\delta_{Ix1}$, $\delta_{Ix2}$, $\delta_{Dx1}$, and $\delta_{Dx2}$ are zero. Based on subplots in Figure 9(1a–1c), the increment of the value of γ results in the decrement of tracking error $E_{y}$, rise and set time, and in the increment of regulation overshoots, but the tracking error in the steady-state remains constant. This confirms that the coefficient γ has the property of the gain $K_{P}$ of the proportional term in the standard PID controller. Whereas the increment of $\delta_{Iy}$ value progressively reduces the steady-state error in time (Figure 9(2a–2c), simultaneously it increases the magnitude and frequency of overshoots after the appearance of gusts. Therefore, coefficients $\delta_{Iy}$ and $\delta_{Iz}$ have the same meaning as the gain $K_{I}=\frac{K_{P}}{T_{I}}$ of the integral term of PID, and they are inversely proportional to the time constant $T_{I}$. Finally, the increment of $\delta_{Dy}$ value reduces the magnitude and the frequency of overshoots like the derivative term of the standard PID does. The role of the derivative term is to react and suppress rapid changes of position tracking errors. Therefore, coefficients $\delta_{Dy}$ and $\delta_{Dz}$ are identical to gains of the derivative term of PID, i.e., $K_{D}=K_{P}\cdot T_{D}$, and they are proportional to the time constant $T_{D}$. Comparing Figure 9(1a) with Figure 9(3c) proves the effectiveness of the nonlinear PID controller based on AAPF. There is a significant improvement in the response of the position tracking control to a lateral wind gust by a conspicuous reduction of the position tracking error, also in the steady-state, the decrement of rising and setting time, and the minimization of overshoots. Because the nonlinear PID controller uses the AAPF as the base, even large values of discussed coefficients will not cause instability and therefore PID tuning by Ziegler–Nicholson method is not possible in this case. Figure 10 presents results for the second scenario which assumes a sequence of gusts in the longitudinal direction relative to the flight path. For the control in the lateral direction, it uses values of coefficients γ, $\delta_{Iy}$, $\delta_{Iz}$, $\delta_{Dy}$, $\delta_{Dz}$ from Figure 9(3c). Time plots of tracking error in this figure allow formulating conclusions that are identical as it is in the case of lateral gusts. Coefficients α and β are gains of the proportional terms of the nonlinear PID, $\delta_{Ix1}$, $\delta_{Ix2}$ are gains of the integral terms, and $\delta_{Dx1}$, $\delta_{Dx2}$ are gains of the derivative terms, respectively for the deceleration zone and the acceleration zone according to the asymmetry of the velocity vector field $V_{i}^{S}\left(x,y,z\right)$ (Figure 1). Gains of the proportional terms minimize the tracking error and rising and setting time, while gains of the integral terms minimize the steady-state error but increase overshoots, and gains of derivative terms decrease overshoot magnitude and frequency.

**Figure 11:**
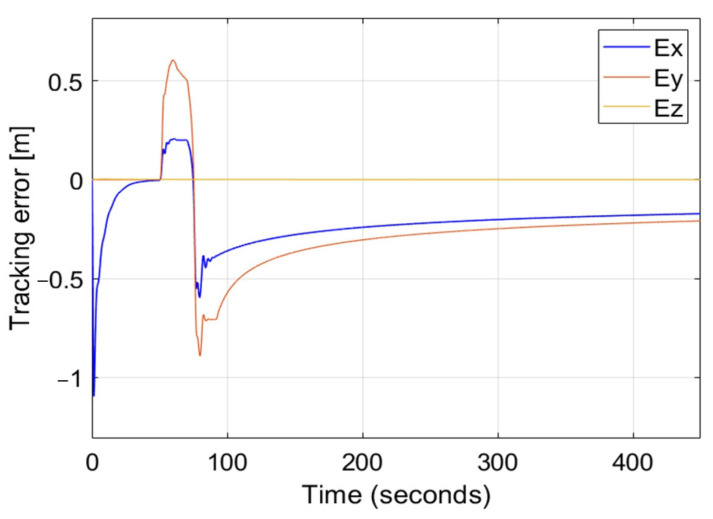
Time plots of position tracking errors for the nonlinear PID controller in the case of simultaneous longitudinal and lateral wind gusts. Applied values of coefficients are as follows: α=10, β=10, γ=10, $\delta_{Ix1}$ = $\delta_{Ix2}$ = $\delta_{Iy}$ = $\delta_{Iz}$ = 1 and $\delta_{Dx1}$ = $\delta_{Dx2}$ = $\delta_{Dy}$ = $\delta_{Dz}$ = 10.

Because there is an interdependence between the lateral and the longitudinal controls observed as oscillations of $E_{x}$ when lateral gusts appear in Figure 9, it is necessary to verify the response of the control in the case of simultaneous gusts in both lateral and longitudinal directions. The sequence of gusts and their speeds are identical to what was in the previous scenarios. Figure 11 presents time plots of tracking errors for such a situation while it uses the nonlinear PID controller with coefficients α=10, β=10, γ=10, $\delta_{Ix1}$ = $\delta_{Ix2}$ = $\delta_{Iy}$ = $\delta_{Iz}$ = 1 and $\delta_{Dx1}$ = $\delta_{Dx2}$ = $\delta_{Dy}$ = $\delta_{Dz}$=10. There are overshoots at the 70th second of simulation in comparison to plots from Figure 9(3c) and Figure 10(3c), but generally, the tracking error does not exceed 1 m and it decreases smoothly in time. This confirms the effectiveness of the control and the validity of the extension of AAPF with integral and derivative PID terms, making position tracking control for nonholonomic UAVs more stable and robust to environmental disturbances.

## 7. Conclusions

**Figure 12:**
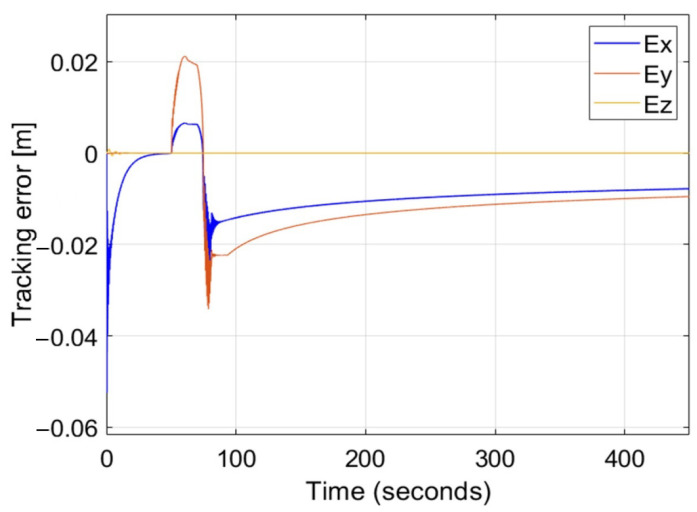
Time plots of position tracking errors for the nonlinear PID controller in the case of simultaneous longitudinal and lateral wind gusts while gains of the PID’s terms are greater than 10,000.

The asymmetrical potential function fulfills requirements for the Lyapunov functions, therefore it provides asymptotical stability in the control of position tracking by nonholonomic, fixed-wing UAVs in applications such as formation flight or target tracking [20]. However, the approach based on the AAPF is ineffective in the case of disturbances that can cause steady-state errors and overshoots. The idea of this work is to extend the definition of the AAPF with the integral and derivative terms to define the novel form of nonlinear PID control loop for the position tracking control by nonholonomic UAVs. The proposed nonlinear PID control loop inherits properties of AAPF, thus it also guarantees the asymptotical stability, and additionally, it gets new properties, enabling the possibility to reduce the steady-state error by the proposed form of the integral terms and to minimize overshoots by the derivative terms. Results of numerical simulations prove the effectiveness of the nonlinear PID controller in improving the control response. The increment of gains of each term gives benefits that are typical for the standard PID controller, i.e., the gain of proportional term minimizes error, rise, and setting time but increases oscillations’ amplitude and frequency, and the gain of integral term minimizes the steady-state error, but increases clearly the amplitude and frequency of oscillations, the gain of derivative term decreases overshoots and oscillations significantly. Because the proportional term results from AAPF, it is not possible to induce system oscillation required to tune the PID loop according to the Ziegler–Nicholson approach. This means that independently of gains of the nonlinear PID loop, the position tracking control remains stable, disturbed only with damped high-frequency oscillations limited by inertia and performance of actuators, which can cause their damage (Figure 12). Similar combinations are applicable for other artificial potential functions having properties of the Lyapunov function, designed for other applications where the precision and the stability of control are highly expected.

## Figures and Tables

**Table 1:** List of symbols and acronyms.

Symbol	Description
UAV	Unmanned aerial vehicle
APF	Artificial potential field
AAPF	Asymmetrical artificial potential field
PID()	Proportional integral derivative controller (function of *x*, *y*, or *z*)
P()	Proportional term (function of *x*, *y*, or *z*)
I()	Integral term (function of *x*, *y*, or *z*)
D()	Derivative term (function of *x*, *y*, or *z*)
PI()	Proportional–integral term (function of *x*, *y*, or *z*)
$V_{L}$	Tracked point velocity
α,β,γ	Gain coefficients of AAPF (proportional term)
$D(\dot{\Psi_{L}})$	Rotation matrix of angular rate of heading angle
ε	Gain coefficient of rotation matrix
$\dot{\Psi_{L}}$	Angular rate of heading angle of tracked point
$U_{i}^{S}$	Asymmetrical artificial potential function
$\nabla U_{i}^{S}$	Gradient of artificial potential function
$V_{i}^{S}$	Velocity vector field
$\delta_{Ix1}$, $\delta_{Ix2}$	Gain coefficients of integral term for *x*
$\delta_{Iy}$	Gain coefficient of integral term for *y*
$\delta_{Iz}$	Gain coefficient of integral term for *z*
$\delta_{Dx1}$, $\delta_{Dx2}$	Gain coefficients of derivative term for *x*
$\delta_{Dy1}$, $\delta_{Dy2}$	Gain coefficients of derivative term for *y*
$\delta_{Dz1}$, $\delta_{Dz2}$	Gain coefficients of derivative term for *z*

**Table 2:** Coefficient list for position tracking error plots from Figure 9.

Controller (Subfigure)	α	β	γ	ε	$\delta_{\mathrm{Iy}}$	$\delta_{\mathrm{Iz}}$	$\delta_{\mathrm{Dy}}$	$\delta_{\mathrm{Dz}}$
P (1a)	10.0	10.0	0.01	0.3	0.0	0.0	0.0	0.0
P (1b)	10.0	10.0	1	0.3	0.0	0.0	0.0	0.0
P (1c)	10.0	10.0	10.0	0.3	0.0	0.0	0.0	0.0
PI (2a)	10.0	10.0	1	0.3	0.1	0.1	0.0	0.0
PI (2b)	10.0	10.0	1	0.3	1.0	1.0	0.0	0.0
PI (2c)	10.0	10.0	1	0.3	10.0	10.0	0.0	0.0
PID (3a)	10.0	10.0	1	0.3	1.0	1.0	0.1	0.1
PID (3b)	10.0	10.0	1	0.3	1.0	1.0	1.0	1.0
PID (3c)	10.0	10.0	1	0.3	1.0	1.0	10.0	10.0

**Table 3:** Coefficient list for position tracking error plots from Figure 10.

Controller (Subfigure)	α	β	γ	ε	$\delta_{\mathrm{Ix}1}$	$\delta_{\mathrm{Ix}2}$	$\delta_{\mathrm{Dx}1}$	$\delta_{\mathrm{Dx}2}$
P (1a)	0.1	0.1	10.0	0.3	0.0	0.0	0.0	0.0
P (1b)	1.0	1.0	10.0	0.3	0.0	0.0	0.0	0.0
P (1c)	10.0	10.0	10.0	0.3	0.0	0.0	0.0	0.0
PI (2a)	1.0	1.0	10.0	0.3	0.1	0.1	0.0	0.0
PI (2b)	1.0	1.0	10.0	0.3	1.0	1.0	0.0	0.0
PI (2c)	1.0	1.0	10.0	0.3	10.0	10.0	0.0	0.0
PID (3a)	1.0	1.0	10.0	0.3	1.0	1.0	0.1	0.1
PID (3b)	1.0	1.0	10.0	0.3	1.0	1.0	1.0	1.0
PID(3c)	1.0	1.0	10.0	0.3	1.0	1.0	10.0	10.0

## Data Availability

Not applicable.
